# Revised definition of predicted left ventricular mass using ambulatory blood pressure in healthy Korean adults

**DOI:** 10.1186/s40885-017-0064-2

**Published:** 2017-04-16

**Authors:** Bae Keun Kim, Enshi Xu, Bo Youl Choi, Yonggu Lee, Soon Gil Kim, Yukio Yamori, Jinho Shin

**Affiliations:** 1 Division of Cardiology, Department of Internal Medicine, Sung Ae General Hospital, Seoul, South Korea; 2 Department of Internal Medicine, Hanyang University College of Medicine, 222 Wangsimni-ro, 04763 Seongdong-gu, Seoul South Korea; 3 Department of Preventive Medicine, Hanyang University College of Medicine, Seoul, South Korea; 4 Institute for World Health Development, Mukogawa Women’s University, Nishinomia, Japan

**Keywords:** Left ventricular hypertrophy, Ambulatory blood pressure monitoring, Stroke work

## Abstract

**Background:**

Left ventricular hypertrophy is influenced by both hemodynamic and non-hemodynamic factors. Ambulatory blood pressure is correlated with left ventricular hypertrophy. We established the influences of hemodynamic and non-hemodynamic factors, including ambulatory blood pressure, on variation in left ventricular mass in healthy Korean adults.

**Method:**

We included 172 subjects (male = 71, female = 101), with normal body mass index and blood pressure, in an analysis of data from the Yangpyung and Yeoju cohort studies and a tertiary cardiovascular center. Left ventricular mass was calculated using the equation: [1.04 × (IVSd + LVDd + PWTd) ^3^-(LDVs^3^)] × 0.8 + 0.6. Stroke volume was calculated (mL/beat) using Teichholz’s formula. Stroke work (SW in gram-meters/beat [g-m/beat]) was computed as ambulatory systolic BP × stroke volume × 0.0144.

**Results:**

Stroke work was the most important determinant associated with left ventricular mass (adjusted *R* ^2^ = 0.442, *p* < 0.001), independent of height^2.7^ and sex. In a regression model including stroke work, height,^2.7^ and sex, the left ventricular mass was predicted by the equation: 43.11 + 0.61 × SW (g-m/beat) + 9.21 × height^2.7^-13.99 × sex (male = 1, female = 2) (constant = 43.11 ± 25.88, adjusted R^2^ = 0.532, *p* < 0.001).

**Conclusion:**

We examined ambulatory blood pressure, as in previous studies, and identified stroke work, height^2.7^, and sex as important determinants of left ventricular mass in Korean adults of normal weight and normal blood pressure. Ambulatory blood pressure is superior to clinical blood pressure for determining stroke work and predicted left ventricular mass.

## Background

Left ventricular hypertrophy (LVH) is a strong independent risk factor of cardiovascular (CV) mortality in hypertensive patients [1]. Left ventricular (LV) mass is influenced by hemodynamic factors, such as high blood pressure (BP) and stroke work, as well as non-hemodynamic factors [2]. However, a number of patients exhibit levels of LV mass that exceed those needed to sustain hemodynamic load, a condition that has been termed as inappropriately high left ventricular mass (iLVM) [3].

iLVM is related to worse CV mortality than appropriate LV mass (aLVM) regardless of the presence of LVH [2]. iLVM may be explained by several mechanisms, including the presence of a higher central BP load, which cannot be explained by brachial BP derived resting stroke work [3], imbalance between growth-promoting factors and growth inhibitory factors [4–6], and BP variability [7]. Genetic factors may also be responsible for exaggerated or overcompensating hypertrophy in response to pressure load [8]. However, the pathogenic mechanism of iLVM is not fully understood [9]. The presence of hidden higher BP load outside of the clinic setting or during sleep that cannot be explained by clinical BP deserves attention.

Considering these probable mechanisms, ambulatory BP is a possible method for further explaining iLVM. No previous studies have examined the utility of ambulatory BP for predicting LV mass. Therefore, we performed this study to establish the influences of hemodynamic and non-hemodynamic factors, including ambulatory BP, on LV mass in healthy Korean adults and to improve our understanding of the mechanisms of iLVM.

## Methods

### Study sample and design

We included 172 (male = 71, female = 101) clinically normal adults of blood pressure below 140/90 mmHg ﻿and normal body mass index (BMI) between 18.5 and 24.9 kg/m^2^ in this study. Of these, 54 were drawn from the ongoing Yangpyung and Yeoju Epidemiologic Cohort Survey between January 1, 2014 and June 30, 2015, and 118 were normotensive subjects visiting the Cardiology Center at Hanyang University Hospital, Seoul, Korea from January 1, 2014 to June 30, 2015. The Yangpyeong and Yeoju Epidemiologic Cohort Survey is a longitudinal, community-based cohort study that was launched in 2007 to examine the etiology of CV diseases, supported by the Korea Centers for Disease Control and Prevention.

Height, weight, clinical BP, and heart rate were measured during the study period before ABPM and echocardiography were performed. The study protocol was approved by the Institutional Review Board (IRB) of Hanyang University Medical Center, Seoul.

### Blood pressure determination

Clinical BP was measured as the average of at least 3 measurements by a physician or qualified nurse using a mercury sphygmomanometer. Ambulatory BP was recorded using a TM-2421 blood pressure monitor (A&D, Saitama, Japan), a device that has undergone independent validation [10, 11]. The device was applied to the non-dominant arm for 24 h. BP was measured every 15 min during the day and every 30 min at night (10 PM to 6 AM). The subjects were instructed to perform their ordinary activities during monitoring and to stay calm when sensing cuff inflation. Daytime and nighttime were recorded individually according to each subject’s self-reported data.

### Echocardiography

Echocardiography was performed on each subject by a single sonographer (JS) using a commercially available machine (iE33; Philips Medical Systems, Andover, MA, USA) with a 1–5 MHz transducer. Mensurements of LV dimensions were performed at or just below the mitral valve tips by the leading edge–to–leading edge method, according to American Society of Echocardiography recommendations with two-dimensional and guided M-mode echocardiogram [12]. LV mass was calculated by the following equation: [1.04 × (IVSd + LVDd + PWTd) 3-LVDd3] × 0.8 + 0.6 [13]. LV end-systolic, end-diastolic, and stroke volume (SV) were calculated by Teichholz’s method. Stroke work (SW) is estimated as systolic BP times stroke volume and is converted in gram-meters (g-m) by multiplying by 0.0144 (SW = systolic BP × SV × 0.0144) [14].

### Statistical analysis

Primary variables were adjusted due to demographic differences between subjects selected from cohort surveys and from hospital patients according to the following procedures. Each subject was called as a dummy variable by assigning the state of 1 to hospital subjects and 2 to cohort subjects. Primary echocardiographic measurements (end-diastolic and end-systolic LV internal dimensions and wall thickness), BP, and heart rate were related to dummy variables independent of age and sex [14, 15]. The variables considered in this qualifying test were therefore adjusted by linear regression analysis (b). Thus, for the adjusted variables (adjV) was adjV = V − b (x − μ), where V was the observed value of the dependent variable, x was the dummy variable representing the group, and μ was the average value of the variable representing the group [16].

Data were expressed as frequencies and percentages for qualitative variables and as the mean ± standard deviation (SD) for quantitative variables. Differences in continuous variables between male and female subjects were assessed with unpaired two-sample *t*-test. Stepwise multiple regression analysis was used to study the hemodynamic and non-hemodynatic predictors of LV mass, with F to enter and F to remove set to *P* < 0.05 and to *P* < 0.10, respectively. Sex was treated as a dummy variable, by assigning 1 to male subjects and 2 to female subjects. Values of 2-tailed *p* < 0.05 were considered statistically significant. Data were analyzed using the Statistical Package for Social Sciences (SPSS) 19.0 software (SPSS Inc., Chicago, IL, USA).

## Results

### General characteristics of the subjects

General characteristics of male versus female subjects are listed in Table 1. Among the 172 subjects, 71 (41.9%) were male and 101 (58.1%) were female. In a comparison of general characteristics between male and female subjects, primary echocardiographic measurements, SV, SW, and LV mass were higher in male subjects. However, LV mass index was not different between male and female subjects.

**Table 1:** Demographic and hemodynamic parameters in normal individuals

	Male (*n* = 71)	Female (*n* = 101)	*p*
Age (years)	46.8 ± 16.2	48.1 ± 11.2	0.565
Height (cm)	171.0 ± 6.1	157.3 ± 5.8	<0.001
Weight (cm)	65.9 ± 6.1	54.3 ± 5.1	<0.001
BMI	22.5 ± 1.6	22.0 ± 1.7	0.031
Clinical SBP (mmHg)	125.9 ± 15.5	122.3 ± 19.1	0.188
Clinical DBP (mmHg)	78.0 ± 9.7	73.6 ± 12.6	0.011
HR (bpm)	68.9 ± 12.8	69.1 ± 9.9	0.913
Ambulatory BP			
24 h SBP (mmHg)	120.7 ± 6.9	115.8 ± 9.3	<0.001
24 h DBP (mmHg)	74.1 ± 5.2	72.6 ± 5.8	0.092
Daytime SBP (mmHg)	122.1 ± 15.7	117.2 ± 14.2	0.033
Daytime DBP (mmHg)	75.1 ± 10.5	73.9 ± 8.9	0.460
Nighttime SBP (mmHg)	112.4 ± 13.2	108.3 ± 14.7	0.063
Nighttime DBP (mmHg)	68.4 ± 7.4	67.0 ± 9.6	0.287
Echocardiography			
IVSTd (cm)	0.9 ± 0.1	0.8 ± 0.1	<0.001
LVDd (cm)	5.0 ± 0.4	4.6 ± 0.4	<0.001
PWTd (cm)	0.8 ± 0.1	0.7 ± 0.1	<0.001
LVDs (cm)	3.3 ± 0.3	3.0 ± 0.3	<0.001
Stroke volume (mL)	75.5 ± 13.0	63.3 ± 11.4	<0.001
Clinical SW (g-m/beat)	137.0 ± 32.1	111.1 ± 27.0	<0.001
Ambulatory SW (g-m/beat)	131.4 ± 24.6	105.8 ± 22.3	<0.001
LV mass (gram)	148.4 ± 29.4	111.0 ± 24.2	<0.001
LV mass index (g/m^2.7^)	34.9 ± 7.0	32.9 ± 8.0	0.087

*BMI* Body mass index, *SBP* systolic blood pressure, *DBP* diastolic blood pressure, *IVSTd* end-diastolic interventricular septal thickness, *LV* left ventricular, *LVDd* end-diastolic left ventricular dimension, *LVDs* end-systolic left ventricular dimension, *PWTd* end-diastolic posterior wall thickness, *SW* Stroke work, *LV* left ventricular

### Effects of age on the relationship between LV mass and body size

**Fig. 1:**
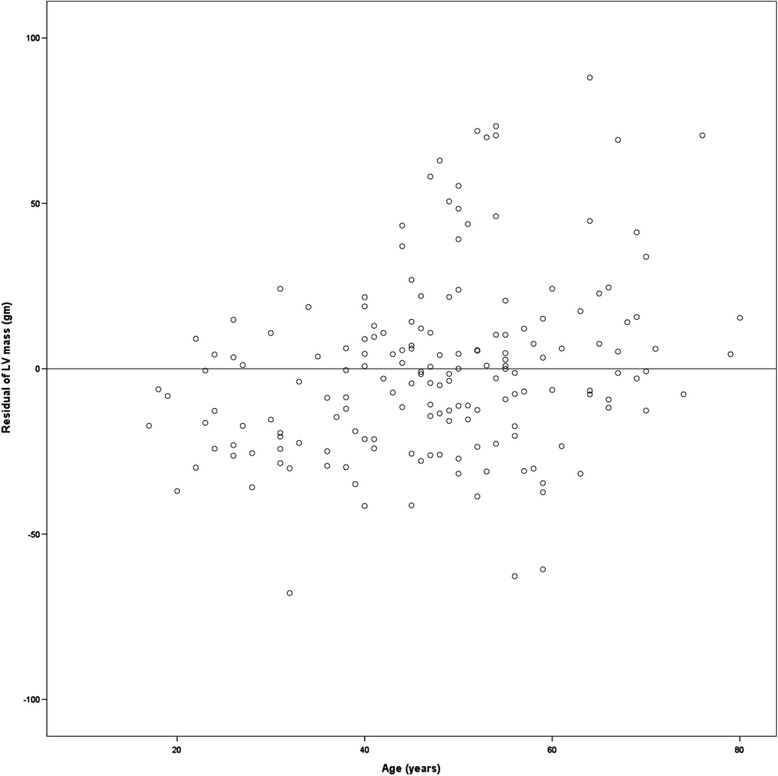
Relationships between age and unstandardized residuals of regression between left ventricular mass and height2.7 in all subjects. The dispersion of residuals were stable with age (homoscedastic distribution)

LV mass was related to all measures of body size, and was related linearly to height2.7. Similar to the results of a previous study [16, 17], residuals of the relationship between LV mass and height2.7 were stable in all subjects (Fig. 1).

### Equation for LV mass predicted by multiple regression analysis of stroke work calculated using ambulatory blood pressure

In a stepwise multiple regression analysis, the variance of LV mass associated with independent variables increased from the 44.2% observed for the univariate relation with ambulatory SW to 53.2% in a multiple regression model including ambulatory SW in all subjects (slope = 0.61, β = 0.50, *p* < 0.001; constant = 43.41, *p* = 0.096), height2.7 (slope = 9.21, β = 0.16, *p* = 0.049) and sex (slope = −21.51, β = −0.33, *p* < 0.001) (Tables 2 and 3).

**Table 2:** Stepwise multiple regression model regarding the factor associated with left ventricular mass

Model	*R*	*R* ^2^	Adjusted *R* ^2^
aSW	0.668	0.446	0.442
aSW, Sex	0.728	0.530	0.524
aSW, Sex, Height^2.7^	0.735	0.540	0.532
cSW	0.613	0.376	0.372
cSW, Sex	0.709	0.503	0.497
cSW, Sex, Height^2.7^	0.718	0.516	0.507

*aSW* stroke work calculated using ambulatory BP, *cSW* stroke work calculated using clinical BP

**Table 3:** Multiple regression model for left ventricular mass

Factor	Slope	beta	*p*
Constant	43.41		0.095
aSW	0.61	0.51	<0.001
Sex	−13.99	−0.33	<0.011
Height^2.7^	9.21	0.16	0.049

*aSW* stroke work calculated using ambulatory BP

### Equation for LV mass predicted by multiple regression analysis of stroke work calculated using clinical blood pressure

In a stepwise multiple regression analysis, the variance of LV mass associated with independent variables increased from the 37.2% observed for the univariate relation with clinical SW to 50.7% in a multiple regression model including clinical SW in all subjects (slope = 0.45, β = 0.44, *p* < 0.001; constant = 61.79, *p* = 0.019), height2.7 (slope = 9.89, β = 0.17, *p* = 0.039) and sex (slope = −17.25, β = −0.27, p = 0.002) (Tables 2 and 4).

**Table 4:** Multiple regression model for left ventricular mass

Factor	Slope	beta	*p*
Constant	61.79		0.019
cSW	0.45	0.53	<0.001
Sex	−17.25	−0.39	0.002
Height^2.7^	9.89	0.17	0.039

*cSW* stroke work calculated using clinical BP

## Discussion

We conducted the present study to further our understanding of predicted LV mass. The main finding of our study is that ambulatory BP is better than clinical BP for predicting LV mass (Table 2). Hemodynamic factors such as stroke work are the most important factors for predicting LV mass.

LV mass is considered a chronic geometric adaptation to cardiac workload that varies over time. Estimating chronic LV load in a single measurement of cardiac workload at rest in time is too difficult. No previous studies have ever used ambulatory BP for predicting LV mass [16, 17] . A previous study showed that resting systolic BP was at least as closely correlated with LV mass as was waking ambulatory systolic BP in normal subjects. The potential imprecision of a single point measurement may be balanced by the size of the study sample [16]. Therefore, ambulatory BP was used instead of clinical BP in this study as a surrogate for mean LV systolic pressure to calculate SW.

Prediction of inappropriate LV mass generally precludes the consideration of sex differences, body size, and the effects of SW because these variables are included in a prediction equation derived from subjects of normal body weight and normal blood pressure [18]. SW is given by systolic BP x stroke volume and is converted to gram-meters (g-m) by multiplying by 0.0144. The increased accuracy of BP measurements by ABPM may strengthen the association between predicted LM mass and BP, mainly by excluding the white-coat and masked (reverse white-coat) effects. For example, if BP is measured in patients with the white-coat effect, the predicted LV mass is often overestimated. In contrast, predictions of LV mass in patients with masked effects tend to be underestimated.

In a regression analysis using clinical BP, the equation for predicting LV mass was as follows: 61.79 + 0.45 × SW (g-m/beat) + 9.89 × height2.7-17.25 × sex (male = 1, female = 2) (constant = 61.79 ± 26.02, adjusted R2 = 0.507, *p* < 0.001). The 50.7% of variation of LV mass is explained by this equation. On the other hand, in a regression model using ambulatory BP, the equation for predicted LV mass was as follows: 43.11 + 0.61 × SW (g-m/beat) + 9.21 × height2.7-13.99 × sex (male = 1, female = 2) (constant = 43.11 ± 25.88, adjusted R2 = 0.532, *p* < 0.001). The 53.2% of variation of LV mass is explained by that equation. In comparison with a previous study using clinical BP, the variation of LV mass is better explained by this equation using ambulatory BP. SW was the most important determinant of LV mass. Therefore, ambulatory BP is better than clinical BP for determining SW and predicting LV mass.

Previous studies suggested that the equation for predicting LV mass should be: 55.37 + 6.64 × height (m2.7) + 0.64 × SW (g-m/beat)-13.2 × sex (male = 1, female = 2) or 54.9 + 7.62 × height (m2.7) + 0.67 × SW (g-m/beat)-13.2 × sex (male = 1, female = 2). In our regression anaylsis including "ambulatory" SW, height (m2.7) and sex, LV mass was predicted by the equation: 43.11 + 9.21 × height2.7 + 0.61 × SW (g-m/beat) -13.99 × sex (male = 1, female = 2).

Similar to previous studies, SW was the most important variable determining LV mass in the present study. LV mass remained stable with age (Fig. 1). Other findings of our study were superior to those of previous studies. Variation in LV mass that remained unexplained could be due to methodological errors, undetectable biological mechanism, and environmental or genetic effects [16, 19]. The extent of technical error may have been reduced by using measurements of ambulatory BP to calculate SW in the present study.

There are limitations to our study. The details of the predicted LV mass equation may not be accurate due to the relatively small sample size and demographic characteristics of the patient population that we included, despite our attempts at statistical adjustment.

## Conclusions

In this study, we demonstrated significant impacts of hemodynamic load as estimated by SW on predictions of LV mass. We found that ambulatory BP is superior to clinical BP for determining the SW and predicting LV mass. Therefore, our new regression equation for predicted LV mass can be a useful tool for evaluating appropriate LV mass in a number of diseases.

## Abbreviations

aLVM: Appropriate left ventricular mass
BMI: Body mass index
BP: Blood pressure
CV: Cardiovascular
iLVM: Inappropriate left ventricular mass
IRB: Institutional review board mass
LV: Left ventricular
LVH: Left ventricular hypertrophy
SD: Standard deviation
SV: Stroke volume
SW: Stroke work
